# Supplementary material for: Cellular Interactions and Formation of an Epithelial “Nanocoating-Like Barrier” with Mesoporous Silica Nanoparticles
Source: Nanomaterials (Basel). 2016 Oct 27;6(11):192. doi: 10.3390/nano6110192 (PMC5245761; doi:10.3390/nano6110192)
Supplement: Supplementary file 1 [file nanomaterials-06-00192-s001.pdf]

Supplementary Materials: Cellular Interactions and Formation of an Epithelial 'Nanocoating-Like Barrier' with Mesoporous Silica Nanoparticles

Xuan Li, Ka Yan Pang, Tsz Wing Ng, Ping Chung Leung, Cheng Fei Zhang, Ken Cham-Fai Leung and Lijian Jin

1. Characterization of the RITC-NPs

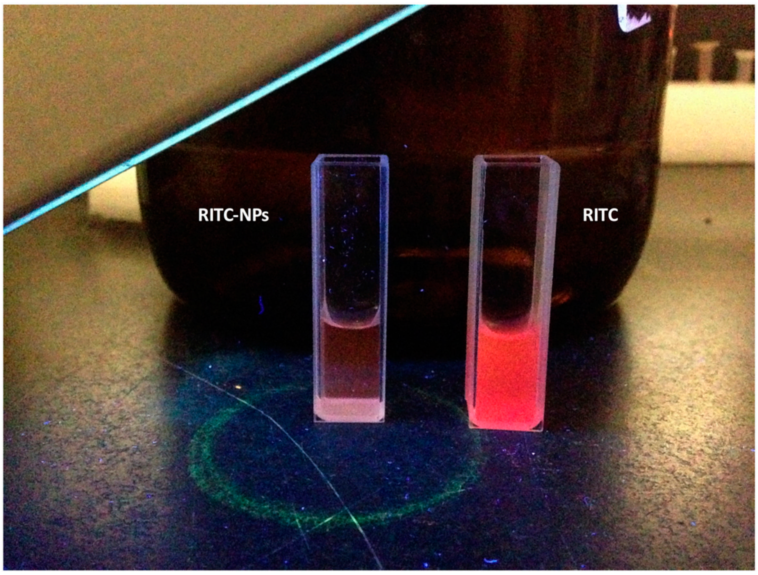

Figure S1. The fluorescence of the RITC-NP dispersion (100 µg/mL) and the RITC ethanol solution (28 µg/mL) under UV excitation.

2. The Histological Profiles of the RHGE and Porcine Ear Skin

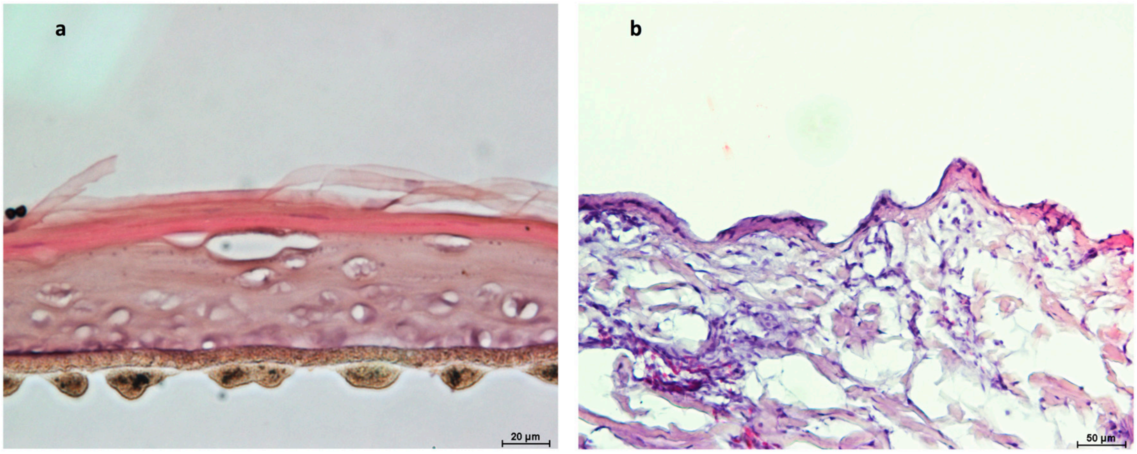

Figure S2. Histological images of the selected epithelial cryosections. Representative sections of RHGE (**a**) and isolated porcine ear skin (**b**) stained with hematoxylin and eosin (H&E) show the stratum corneum layer (red color) and the underlying epithelial cells (dark brown in RHGE and light blue in porcine ear skin).

3. The Presence of RITC-NPs in Hair Follicles of Porcine Ear Skin

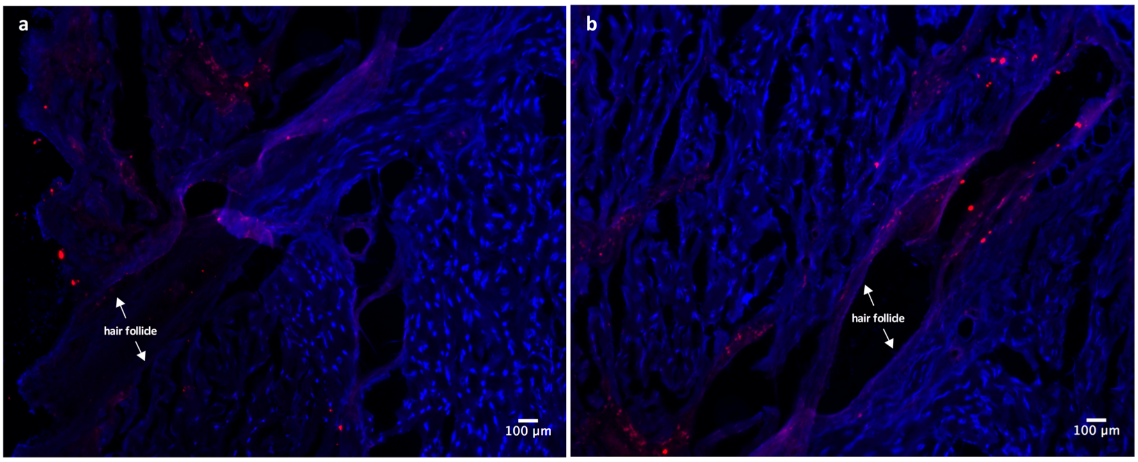

Figure S3. The fluorescent images of the RITC-NPs in upper (**a**) and lower (**b**) parts of a hair follicle of porcine ear skin after 24 h of treatment. The cell nuclei and RITC-NPs were detected by blue and red fluorescence, respectively.
